# Pistas Contraditórias na Hipertrofia Ventricular Esquerda Grave: Uma Apresentação Incomum de Amiloidose ATTR

**DOI:** 10.36660/abc.20250832

**Published:** 2026-06-16

**Authors:** Ana Sofia Nogueira Fernandes, João Faria, Catarina Ferreira Vieira

**Affiliations:** 1 Braga Local Health Unit Braga Portugal Braga Local Health Unit, Braga– Portugal

**Keywords:** Amiloidose, Cardiomiopatias, Síncope, Hipertrofia

## Introdução

Nas últimas décadas, grandes avanços remodelaram a compreensão clínica e epidemiológica da amiloidose cardíaca por transtirretina (ATTR). O progresso na imagem cardiovascular possibilitou a identificação mais precoce e precisa da doença.^1^ Em particular, a cintilografia óssea com traçadores marcados com ^99^mTc simplificou consideravelmente o diagnóstico não invasivo da cardiomiopatia por ATTR, reduzindo substancialmente a necessidade de biópsia endomiocárdica na maioria dos casos.^1^,^2^ Contudo, desafios diagnósticos podem surgir quando os achados clínicos, genéticos e de imagem são discordantes. Relatamos um caso de cardiomiopatia hereditária por ATTR (ATTRv) comprovada por biópsia com cintilografia óssea negativa, ilustrando uma importante armadilha diagnóstica no algoritmo diagnóstico atual.

### Apresentação do caso

Um homem de 72 anos com histórico de hipertensão foi internado após um episódio de síncope sem pródromos, dor torácica ou palpitações. Na admissão, se encontrava hemodinamicamente estável, com bradicardia sinusal (54 bpm). O exame físico e a auscultação cardíaca não apresentaram alterações, e o histórico familiar era irrelevante.

O eletrocardiograma inicial mostrou um atraso de condução intraventricular inespecífico e inversão da onda T nas derivações V2-V5. A troponina I atingiu um pico de 21 ng/ml. Pouco depois da admissão, ele desenvolveu taquicardia ventricular sustentada, causando parada cardíaca, revertida com sucesso por desfibrilação. O ecocardiograma transtorácico revelou hipertrofia ventricular esquerda grave (septo de 19 mm), função sistólica preservada e hipocinesia distal inferior. A angiografia coronária excluiu doença arterial coronariana epicárdica. Dias depois, desenvolveu bloqueio atrioventricular total, necessitando de marca-passo temporário, seguido de recorrência de taquicardia ventricular sustentada tratada com amiodarona. Posteriormente, foi implantado um cardioversor-desfibrilador implantável.

**Figura 1:**
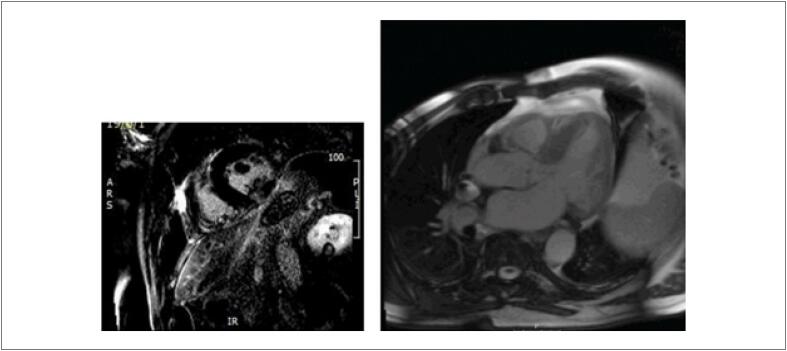
Ressonância magnética cardíaca mostrando hipertrofia ventricular esquerda.

A ressonância magnética cardíaca mostrou hipertrofia septal grave com realce tardio de gadolínio na junção interventricular inferior e uma cicatriz inferolateral, sugerindo inicialmente cardiomiopatia hipertrófica (Figura 1). Considerou-se a possibilidade de doença de Fabry, mas a atividade da α-galactosidase A e o sequenciamento do gene GLA foram normais.

Durante o acompanhamento, o paciente desenvolveu disfunção sistólica progressiva do ventrículo esquerdo, alta carga de estimulação ventricular e piora dos sintomas de insuficiência cardíaca (classe III da NYHA), o que levou à necessidade de terapia de ressincronização cardíaca.

**Figura 2:**
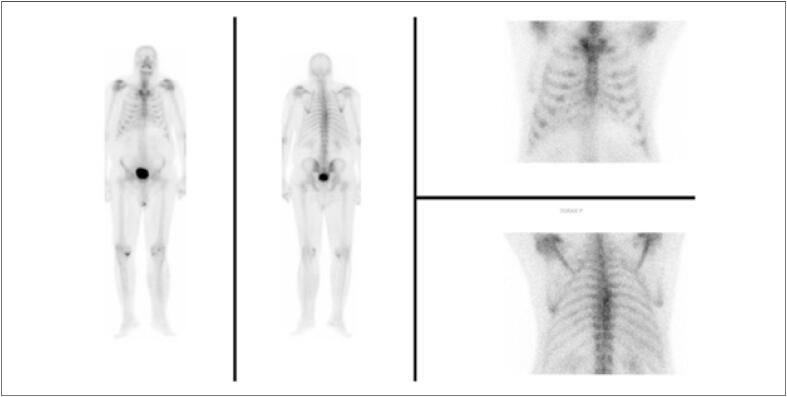
Cintilografia óssea com DPD mostrando um escore de Perugini de 0.

A investigação etiológica descartou amiloidose monoclonal de cadeia leve (AL) (cadeias leves séricas e eletroforese normais). A cintilografia óssea com ^99^mTc-DPD mostrou um escore de Perugini de 0 (Figura 2). O teste genético, no entanto, identificou uma variante heterozigótica da transtirretina (TTR) c.148G>A (p.Val50Met), associada à amiloidose hereditária por transtirretina (ATTR). Diante da discordância entre o resultado genético, o fenótipo clínico e a cintilografia negativa, foi realizada uma biópsia endomiocárdica, que demonstrou fibrose intersticial, hialinização vascular e birrefringência ao vermelho Congo, confirmando a cardiomiopatia por ATTR.

Ele foi encaminhado a um centro especializado e se iniciou o tratamento com tafamidis. Devido ao seu fenótipo cardíaco exclusivo e à ATTR hereditária de início tardio, foi mantido um acompanhamento clínico rigoroso. Recomendou-se o rastreio familiar para permitir a identificação precoce de parentes em risco.

## Discussão

Este caso ilustra um cenário raro, porém clinicamente relevante, de cardiomiopatia ATTRv comprovada por biópsia com cintilografia óssea negativa. Do ponto de vista prático, este caso destaca uma nuance importante dentro do algoritmo diagnóstico contemporâneo para amiloidose cardíaca. Embora a cintilografia óssea com traçadores marcados com ^99^mTc tenha revolucionado o diagnóstico não invasivo da cardiomiopatia ATTR e reduzido significativamente a necessidade de confirmação histológica na maioria dos casos, sua interpretação requer cuidadosa integração com dados clínicos e genéticos.^1-3^ Quando a cintilografia cardíaca é negativa e as cadeias leves monoclonais estão ausentes, a amiloidose cardíaca é geralmente considerada improvável.^1,2,4^ No entanto, se a suspeita clínica persistir — particularmente na presença de achados de imagem sugestivos ou uma variante patogênica do gene TTR — a biópsia endomiocárdica permanece uma ferramenta diagnóstica valiosa e deve ser considerada.^2,3^

Em grandes estudos de validação, a captação miocárdica do traçador com um escore de Perugini ≥2 demonstrou excelente desempenho diagnóstico para cardiomiopatia por ATTR. Em uma coorte multicêntrica utilizando biópsia endomiocárdica como padrão de referência, um escore de Perugini ≥2 apresentou sensibilidade de aproximadamente 90% e especificidade de 95% para cardiomiopatia por ATTR. É importante ressaltar que cerca de 10% dos pacientes com ATTR comprovada por biópsia apresentaram escores de Perugini <2, e o valor preditivo negativo de um escore de Perugini <2 foi de apenas 79%.^5^ Esses achados indicam que a captação negativa ou de baixo grau do traçador não exclui completamente a cardiomiopatia por ATTR, particularmente quando a suspeita clínica permanece alta.

Essas observações destacam que a cintilografia óssea negativa pode ocorrer ocasionalmente mesmo em casos de cardiomiopatia por ATTR comprovada por biópsia, e pode ser explicada por diversos mecanismos fisiopatológicos. A maioria dos estudos de validação da cintilografia óssea inclui predominantemente populações com ATTR do tipo selvagem (ATTRwt), e o desempenho diagnóstico parece menos consistente em ATTRv. A ATTRwt tipicamente apresenta infiltração miocárdica difusa e captação consistente do traçador. Em contraste, a ATTRv abrange um grupo heterogêneo de mutações com padrões variáveis de deposição de amiloide, o que pode levar a uma menor captação miocárdica do traçador e a resultados falso-negativos ocasionais na cintilografia. Padrões cintilográficos variáveis foram descritos em diferentes variantes da TTR, incluindo Val50Met, e uma sensibilidade marcadamente reduzida foi relatada em mutações específicas, como Phe64Leu.^6^ Além disso, nos estágios iniciais da doença, a carga amiloide miocárdica pode ser insuficiente para gerar uma captação significativa do radiotraçador, particularmente quando a deposição ainda é limitada ou irregular. A deposição miocárdica atípica ou focal também pode contribuir para resultados falso-negativos quando a infiltração amiloide é heterogênea em vez de difusa.^7^

Além dos fatores biológicos, os aspectos técnicos e metodológicos da cintilografia óssea também podem influenciar a interpretação. A imagem planar isoladamente pode ser insuficiente para caracterizar com precisão a captação miocárdica, particularmente em casos de baixa intensidade ou distribuição heterogênea do traçador. A integração da SPECT/CT melhora a resolução espacial e permite a diferenciação entre a verdadeira captação miocárdica e a atividade no pool sanguíneo. Variações no tempo de aquisição, na dose do radiotraçador e nos protocolos institucionais podem influenciar ainda mais a qualidade e a interpretação da imagem. Além disso, a variabilidade interobservador, especialmente na distinção entre os graus 0 e 1 de Perugini, pode levar à subnotificação de captação mínima ou focal, particularmente em centros com experiência limitada em imagem de amiloidose cardíaca.^2,3^

A distinção entre ATTRwt e ATTRv também é relevante neste contexto de heterogeneidade diagnóstica. A ATTRwt afeta predominantemente homens idosos e tipicamente se apresenta como um fenótipo cardiomiopático progressivo com envolvimento miocárdico difuso. Em contraste, a ATTRv abrange um amplo espectro de variantes patogênicas com idade de início variável e manifestações extracardíacas. Dependendo da mutação, o quadro clínico pode ser dominado por neuropatia ou doença cardíaca. A mutação Val50Met está classicamente associada a formas neuropáticas de início precoce e é endêmica em Portugal, Suécia e Japão; no entanto, fenótipos de início tardio e predominantemente cardíacos têm sido cada vez mais reconhecidos. Essa variabilidade pode explicar parcialmente perfis de imagem atípicos e ressalta a importância da integração de dados genéticos, clínicos e de imagem no processo diagnóstico. Reconhecer essas distinções tem importantes implicações prognósticas e terapêuticas e é essencial para aconselhamento genético adequado e triagem familiar.^1,2^

Em conjunto, essas considerações biológicas e técnicas reforçam a importância de interpretar os achados da cintilografia dentro de um contexto clínico e genético abrangente, e não como determinantes isolados do diagnóstico. Em ambientes menos especializados, a dependência excessiva em achados cintilográficos isolados pode levar ao fechamento diagnóstico prematuro e à subnotificação de apresentações atípicas.^1,2^ Quando há discordância entre a suspeita clínica, as evidências genéticas e os resultados de imagem, uma avaliação mais aprofundada — preferencialmente em centros especializados com experiência em amiloidose cardíaca — torna-se essencial. Mesmo na era da imagem cardíaca multimodal avançada, a biópsia endomiocárdica permanece o padrão ouro e continua a desempenhar um papel crucial no estabelecimento de um diagnóstico definitivo, na prevenção de erros de classificação e na prevenção de atrasos no início da terapia modificadora da doença.^8^

## Conclusão

Este caso enfatiza que os algoritmos diagnósticos contemporâneos para cardiomiopatia ATTR, embora altamente eficazes, requerem interpretação clínica contextual.^1-3^ O reconhecimento de potenciais discordâncias diagnósticas é essencial para garantir a classificação precisa e o início oportuno da terapia modificadora da doença, particularmente em formas hereditárias com expressão fenotípica heterogênea.^6^

## Data Availability

Os conteúdos subjacentes ao texto da pesquisa estão contidos no manuscrito.
