# Supplementary material for: Pistas Contraditórias na Hipertrofia Ventricular Esquerda Grave: Uma Apresentação Incomum de Amiloidose ATTR
Source: Arq Bras Cardiol. 2026 Jun 16;123(5):e20250832. [Article in Portuguese] doi: 10.36660/abc.20250832 (PMC13399371; doi:10.36660/abc.20250832)
Supplement: Translation

# Translation: Conflicting Clues in Severe LV Hypertrophy: An Unusual Presentation of ATTR Amyloidosis

**DOI:** 10.36660/abc.20250832i

**Authors:** Ana Sofia Nogueira Fernandes, João Faria, Catarina Ferreira Vieira

**Affiliations:** 1 Braga Portugal Braga Local Health Unit,1 Braga - Portugal

**Keywords:** Amyloidosis, Cardiomyopathies, Syncope, Hypertrophy

## Introduction

In recent decades, major advances have reshaped the clinical and epidemiological understanding of transthyretin (TTR) cardiac amyloidosis (ATTR). Progress in cardiovascular imaging has enabled earlier and more accurate identification of the disease.^1^ In particular, bone scintigraphy with ^99^mTc-labeled tracers has markedly simplified the non-invasive diagnosis of ATTR cardiomyopathy, substantially reducing the need for endomyocardial biopsy in most cases.^1,2^ Nevertheless, diagnostic challenges may arise when clinical, genetic, and imaging findings are discordant. We report a case of biopsy-proven hereditary ATTR (ATTRv) cardiomyopathy with negative bone scintigraphy, illustrating an important diagnostic pitfall within the contemporary diagnostic algorithm.

### Case presentation

A 72-year-old man with a history of hypertension was admitted after a syncopal episode without prodromes, chest pain, or palpitations. On admission, he was hemodynamically stable, with sinus bradycardia (54 bpm). Physical examination and auscultation were unremarkable, and family history was irrelevant.

The initial electrocardiogram showed a nonspecific intraventricular conduction delay and T-wave inversion in leads V2–V5. Troponin I peaked at 21 ng/ml. Shortly after admission, he developed sustained ventricular tachycardia, causing cardiac arrest, successfully terminated by defibrillation. Transthoracic echocardiography revealed severe left ventricular hypertrophy (septum 19 mm), preserved systolic function, and distal inferior hypokinesia. Coronary angiography excluded epicardial coronary artery disease. Days later, complete atrioventricular block developed, requiring temporary pacing, followed by recurrent sustained ventricular tachycardia treated with amiodarone. An implantable cardioverter-defibrillator was subsequently implanted.

Cardiac magnetic resonance showed severe septal hypertrophy with late gadolinium enhancement at the inferior interventricular junction and an inferolateral scar, initially suggesting hypertrophic cardiomyopathy (Figure 1). Fabry disease was considered, but α-galactosidase A activity and *GLA* gene sequencing were normal.

**Figure 1:** Cardiac MRI showing left ventricular hypertrophy.

During follow-up, the patient developed progressive LV systolic dysfunction, a high ventricular pacing burden, and worsening heart failure symptoms (NYHA class III), prompting upgrade to cardiac resynchronization therapy.

The etiologic investigation ruled out monoclonal light chain (AL) amyloidosis (normal serum light chains and electrophoresis). Bone scintigraphy with ^99^mTc-DPD showed a Perugini score of 0 (Figure 2). Genetic testing, however, identified a heterozygous transthyretin (TTR) c.148G>A (p.Val50Met) variant, associated with hereditary TTR amyloidosis (ATTR). Given the discordance between the genetic result, clinical phenotype, and negative scintigraphy, an endomyocardial biopsy was performed, demonstrating interstitial fibrosis, vascular hyalinization, and Congo red birefringence, confirming ATTR cardiomyopathy.

**Figure 2:** Bone scintigraphy with DPD showing a Perugini score of 0.

He was referred to a specialized centre, and tafamidis therapy was initiated. Given his exclusive cardiac phenotype and late-onset hereditary ATTR, close clinical monitoring was maintained. Family screening was recommended to enable early identification of at-risk relatives.

## Discussion

This case illustrates a rare but clinically relevant scenario of biopsy-proven ATTRv cardiomyopathy with negative bone scintigraphy. From a practical standpoint, this case highlights an important nuance within the contemporary diagnostic algorithm for cardiac amyloidosis. Although bone scintigraphy with 99mTc-labeled tracers has revolutionized the non-invasive diagnosis of ATTR cardiomyopathy and significantly reduced the need for histological confirmation in most cases, its interpretation requires careful integration with clinical and genetic data.^1-3^ When cardiac scintigraphy is negative and monoclonal light chains are absent, cardiac amyloidosis is generally considered unlikely.^1,2,4^ However, if clinical suspicion persists — particularly in the presence of suggestive imaging findings or a pathogenic TTR variant — endomyocardial biopsy remains a valuable diagnostic tool and should be considered.^2,3^

In large validation studies, myocardial tracer uptake with a Perugini score ≥2 has demonstrated excellent diagnostic performance for ATTR cardiomyopathy. In a multicenter cohort using endomyocardial biopsy as the reference standard, a Perugini score ≥2 showed a sensitivity of approximately 90% and specificity of 95% for ATTR cardiomyopathy. Importantly, around 10% of patients with biopsy-proven ATTR presented with Perugini scores <2, and the negative predictive value of a Perugini score <2 was only 79%.^5^ These findings indicate that negative or low-grade tracer uptake does not completely exclude ATTR cardiomyopathy, particularly when clinical suspicion remains high.

These observations highlight that negative bone scintigraphy may occasionally occur even in biopsy-proven ATTR cardiomyopathy and may be explained by several pathophysiological mechanisms. Most validation studies of bone scintigraphy include predominantly wild-type ATTR (ATTRwt) populations, and diagnostic performance appears less consistent in ATTRv. ATTRwt typically presents with diffuse myocardial infiltration and consistent tracer uptake. In contrast, ATTRv encompasses a heterogeneous group of mutations with variable patterns of amyloid deposition, which may lead to lower myocardial tracer uptake and occasional false-negative scintigraphy. Variable scintigraphic patterns have been described across different TTR variants, including Val50Met, and markedly reduced sensitivity has been reported in specific mutations such as Phe64Leu.^6^ In addition, in early disease stages, myocardial amyloid burden may be insufficient to generate significant radiotracer uptake, particularly when deposition is still limited or uneven. Atypical or focal myocardial deposition may also contribute to false-negative findings when amyloid infiltration is heterogeneous rather than diffuse.^7^

Beyond biological factors, technical and methodological aspects of bone scintigraphy may also influence interpretation. Planar imaging alone may be insufficient to accurately characterize myocardial uptake, particularly in cases of low-grade or heterogeneous tracer distribution. The integration of SPECT/CT improves spatial resolution and allows differentiation between true myocardial uptake and blood pool activity. Variations in acquisition timing, radiotracer dose, and institutional protocols may further influence image quality and interpretation. In addition, interobserver variability, especially in distinguishing Perugini grades 0 and 1, may lead to under-recognition of minimal or focal uptake, particularly in centers with limited experience in cardiac amyloidosis imaging.^2,3^

Distinguishing ATTRwt from ATTRv is also relevant within this context of diagnostic heterogeneity. ATTRwt predominantly affects elderly men and typically presents as a progressive cardiomyopathic phenotype with diffuse myocardial involvement. In contrast, ATTRv encompasses a broad spectrum of pathogenic variants with variable age of onset and extracardiac manifestations. Depending on the mutation, the clinical picture may be dominated by neuropathy or cardiac disease. Val50Met is classically associated with early-onset neuropathic forms and is endemic in Portugal, Sweden, and Japan; however, late-onset and predominantly cardiac phenotypes have been increasingly recognized. Such variability may partially explain atypical imaging profiles and underscores the importance of integrating genetic, clinical, and imaging data in the diagnostic process. Recognizing these distinctions has important prognostic and therapeutic implications and is essential for appropriate genetic counseling and family screening.^1,2^

Taken together, these biological and technical considerations reinforce that scintigraphy findings should be interpreted within a comprehensive clinical and genetic framework rather than as isolated determinants of diagnosis. In less specialized settings, overreliance on isolated scintigraphic findings may lead to premature diagnostic closure and underrecognition of atypical presentations.^1,2^ When discordance arises between clinical suspicion, genetic evidence, and imaging results, further evaluation—preferably in specialized centers with expertise in cardiac amyloidosis—becomes essential. Even in the era of advanced multimodality cardiac imaging, endomyocardial biopsy remains the gold standard and continues to play a critical role in establishing a definitive diagnosis, preventing misclassification, and avoiding delay in the initiation of disease-modifying therapy.^8^

## Conclusion

This case emphasizes that contemporary diagnostic algorithms for ATTR cardiomyopathy, although highly effective, require contextual clinical interpretation.^1-3^ Recognition of potential diagnostic discordance is essential to ensure accurate classification and timely initiation of disease-modifying therapy, particularly in hereditary forms with heterogeneous phenotypic expression.^6^

## Data Availability

The underlying content of the research text is contained within the manuscript.
